# Vacuum-Treated Brown Mesoporous TiO_2_ Nanospheres with Tailored Defect Structures for Enhanced Photoresponsive Properties

**DOI:** 10.3390/molecules30244746

**Published:** 2025-12-12

**Authors:** Yue Gao, Ting Feng, Xuan Qi, Hao Yan, Jinfeng Du, Yu Zhang, Junfeng Zhang

**Affiliations:** 1 College of Chemical and Materials Engineering, Hainan Vocational University of Science and Technology, Haikou 571126, China; 2 School of Materials Science and Engineering, Qingdao University of Science and Technology, Qingdao 266042, China; 3 Hangzhou Xigu Technology Co., Ltd., Hangzhou 310000, China; 4 Liaoning Key Laboratory for Green Synthesis and Preparative Chemistry of Advanced Materials, College of Chemistry, Liaoning University, Shenyang 110036, China; 5 School of Chemistry and Chemical Engineering, Hainan University, Haikou 570228, China

**Keywords:** TiO_2_, photocatalytic, band gap, solar-driven, self-doping

## Abstract

TiO_2_ Nanospheres with a large surface area were synthesized via a hydrothermal reaction using titanium glycolate. The samples were subsequently subjected to different vacuum oven treatment times (2, 4, 6, and 8 h), resulting in Ti^3+^ self-doping. Comprehensive characterization was performed using transmission electron microscopy (TEM), X-ray diffraction (XRD), and X-ray photoelectron spectroscopy (XPS). The synthesized TiO_2_ Nanospheres exhibited significantly enhanced photocurrent and efficient photocatalytic activity under visible light irradiation, demonstrating their potential for applications in solar-driven water splitting. The results highlight the influence of Ti^3+^ self-doping on improving the photoactivity and photosensitivity of the material.

## 1. Introduction

Titanium dioxide (TiO_2_) nanoparticles have garnered significant attention as ideal photocatalysts due to their excellent oxidation capabilities and broad applicability. However, the wide band gap (3.2 eV) of TiO_2_ limits its ability to absorb and utilize visible light, restricting its photocatalytic performance to ultraviolet light with wavelengths shorter than 387 nm, which hinders its practical use in various applications [1,2,3]. To overcome this limitation, considerable efforts have been devoted to enhancing the photocatalytic activity of TiO_2_ through morphological modifications. For instance, synthesizing TiO_2_ in specific morphologies such as rod-like or spherical structures can significantly increase the surface area and light scattering capabilities, improving solar light utilization efficiency. Additionally, substantial research has focused on enhancing TiO_2_’s photocatalytic activity under visible light, including surface defect engineering to modulate its electronic structure and improve light absorption [4,5,6], Among all visible light-absorbing TiO_2_ materials, black TiO_2_ has attracted considerable attention due to its high stability, simple preparation methods, and excellent photocatalytic activity when synthesized through various approaches [7,8,9,10,11,12,13,14].

In recent years, considerable efforts have been made to enhance the photocatalytic performance of TiO_2_, particularly under visible light, by modifying its electronic structure. One of the most studied modifications is the introduction of Ti^3+^ defects, which has been shown to significantly narrow the bandgap, enhancing light absorption. Among the various methods to modify TiO_2_, black TiO_2_ has garnered particular attention. Black TiO_2_, a modified form of TiO_2_ with oxygen vacancies and Ti^3+^ species, has demonstrated superior photocatalytic activity under visible light due to its reduced bandgap and improved light absorption capabilities. For example, Chen et al. successfully synthesized black hydrogenated TiO_2_ nanocrystals, which exhibited remarkable solar absorption enhancement for photocatalysis, achieving a reduction in the bandgap from 3.2 eV to 2.2 eV. Similarly, Yu et al. explored Pt-decorated black TiO_2_ nanotube arrays, demonstrating a synergistic effect between pH-dependent reactions and enhanced photocatalytic performance. Moreover, Zahid et al. employed laser-induced bandgap engineering of black TiO_2_, where the phase transformation from anatase to rutile and the formation of Ti^3+^ defects led to a significant bandgap reduction, further boosting its photocatalytic efficiency for energy applications. These studies underline the critical role of defect engineering in optimizing TiO_2_ for photocatalysis, particularly through the creation of sub-bandgap states and the formation of oxygen vacancies, which facilitate improved charge carrier dynamics [12,15,16].

In comparison, our approach, which utilizes vacuum treatment to introduce Ti^3+^ self-doping and oxygen vacancies, offers a simpler and more scalable method for enhancing the photocatalytic properties of TiO_2_. Unlike other methods that require high temperatures or complex procedures, our approach retains the anatase phase while enhancing photocatalytic efficiency through defect engineering. The results obtained from our study indicate that the Ti^3+^ self-doping and oxygen vacancy formation not only improve the material’s visible light absorption but also increase its photocatalytic activity, as demonstrated by the 4hTiO_2_ sample in our experiments [17,18].

Mingyang Xing and colleagues have employed vacuum high-temperature activation treatments on P25 TiO_2_ at different time intervals, revealing the formation of Ti^3+^ and oxygen vacancies (O_V_) in the treated samples, which led to enhanced photocatalytic performance. Their findings suggest that this simple, cost-effective activation method could serve as a key strategy for expanding the application of photocatalysts [19,20,21]. These advancements highlight the potential for modifying TiO_2_ to improve its photocatalytic activity and sensitivity under visible light.

Inspired by these studies, we hypothesize that mesoporous TiO_2_ Nanospheres, which are capable of absorbing a small portion of solar energy (mainly UV light, which constitutes only 4% of the solar spectrum), can benefit from vacuum high-temperature activation treatment [22,23,24,25]. This treatment may enhance their photosensitivity and photoelectric conversion efficiency, making them more effective for solar-driven applications [20,26,27,28,29,30]. In this study, mesoporous TiO_2_ Nanospheres were treated using vacuum high-temperature activation at different times (2, 4, 6, and 8 h), resulting in 2hTiO_2_, 4hTiO_2_, 6hTiO_2_, and 8hTiO_2_ samples [21,31]. The photocatalytic degradation of Rhodamine B (RHB), photocurrent generation, and water oxidation experiments were conducted to investigate the photo-induced charge properties and photocatalytic performance of these samples. The results were compared to determine the optimal treatment time. Finally, X-ray photoelectron spectroscopy (XPS) and flat band potential measurements were performed to further understand the superior photoelectric properties of the optimal sample.

## 2. Results and Discussion

### 2.1. TEM Analysis

**Figure 1:**
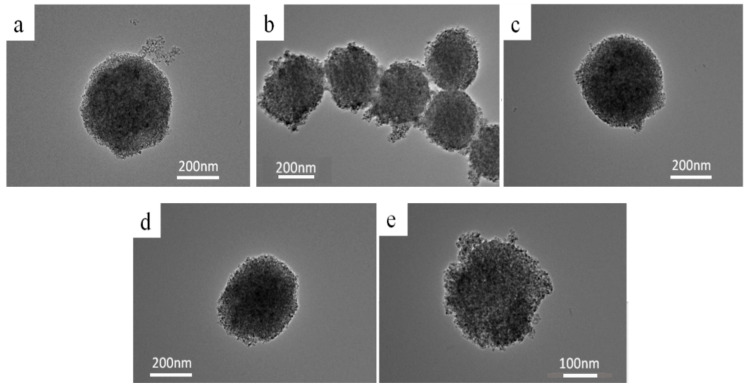
TEM images of samples obtained after vacuum activate for different time. (**a**) TiO_2_; (**b**) 2h TiO_2_; (**c**) 4hTiO_2_; (**d**) 6hTiO_2_; (**e**) 8hTiO_2_.

Figure 1 presents the transmission electron microscope (TEM) images of mesoporous TiO_2_ and TiO_2_ samples treated under high-temperature vacuum conditions. As shown in Figure 1, all five samples exhibit an average particle diameter of approximately 250 nm [32,33], with a rough surface structure that facilitates the scattering of incident solar light, thereby enhancing light utilization. The structures are relatively loose, with numerous pores present on the surface, which significantly increases the surface area of the nanospheres.

The structural morphology of the four activated samples (2hTiO_2_, 4hTiO_2_, 6hTiO_2_, and 8hTiO_2_) remains largely similar to that of the untreated TiO_2_, with only slight signs of sintering observed. This suggests that the vacuum high-temperature treatment did not significantly alter the microscopic morphology of the samples. The modest sintering effect indicates that the activation process primarily affects the electronic properties of the material rather than causing significant structural changes.

These results suggest that the vacuum treatment time does not dramatically influence the overall morphology but may lead to subtle modifications in the surface properties and defects, which could play a critical role in enhancing the photocatalytic activity and photoelectric performance of the TiO_2_ Nanospheres. Further investigation into the relationship between surface characteristics and photocatalytic efficiency is necessary to identify the optimal treatment conditions for improved solar-driven applications.

### 2.2. XRD Analysis

**Figure 2:**
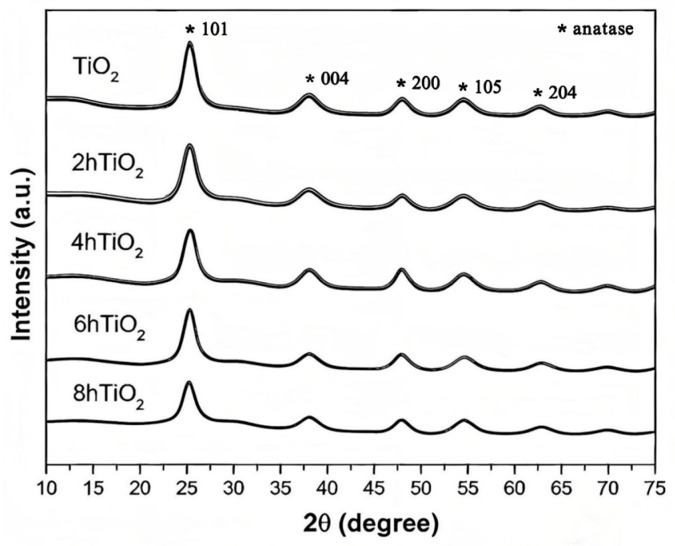
XRD patterns of mesoporous TiO_2_ Nanospheres and 2h TiO_2_,4h TiO_2_,6h TiO_2_,8h TiO_2_.

Figure 2 shows the X-ray diffraction (XRD) patterns of TiO_2_, 2hTiO_2_, 4hTiO_2_, 6hTiO_2_, and 8hTiO_2_. The mesoporous TiO_2_ Nanospheres exhibit characteristic diffraction peaks at 2θ values of 25.5°, 37.9°, 48.2°, 54.1°, and 63.1°, corresponding to the typical anatase phase of TiO_2_. Which correspond to the typical anatase phase of TiO_2_. Specifically, the primary peaks at 25.5° (101), 37.9° (004), and 48.2° (200), along with the secondary peaks at 54.1° (105) and 63.1° (204), confirm the presence of anatase TiO_2_ in all treated samples. These findings indicate that the treatment did not induce a phase transition, but rather affected the degree of crystallization, which influences the photocatalytic properties [34]. The XRD patterns of the treated samples (2hTiO_2_, 4hTiO_2_, 6hTiO_2_, and 8hTiO_2_) show diffraction peaks that are consistent with the TiO_2_ anatase phase, with no significant phase transformation observed.

Upon comparing the main peaks with standard reference patterns, it is evident that all five samples exhibit the anatase phase, though the intensity of the peaks decreases progressively with increasing treatment time. Notably, the intensity reduction is more pronounced in the 8hTiO_2_ sample, indicating that the vacuum high-temperature treatment leads to a decrease in the crystallinity of TiO_2_, rather than altering its crystal structure. This suggests that while the treatment does not induce a phase transition, it does impact the degree of crystallization, which may influence the material’s photocatalytic properties by increasing the number of surface defects and altering electron transport characteristics.

These findings indicate that the vacuum high-temperature treatment primarily affects the crystallinity of TiO_2_, and the decrease in peak intensity with treatment time is consistent with the expected reduction in crystallite size or the formation of amorphous regions. Therefore, appropriate heat treatment can generate suitable doping states and enhance photocatalytic activity, while excessive heat treatment time may lead to the formation of other phases, thereby reducing the activity.

### 2.3. UV-Vis Analysis

**Figure 3:**
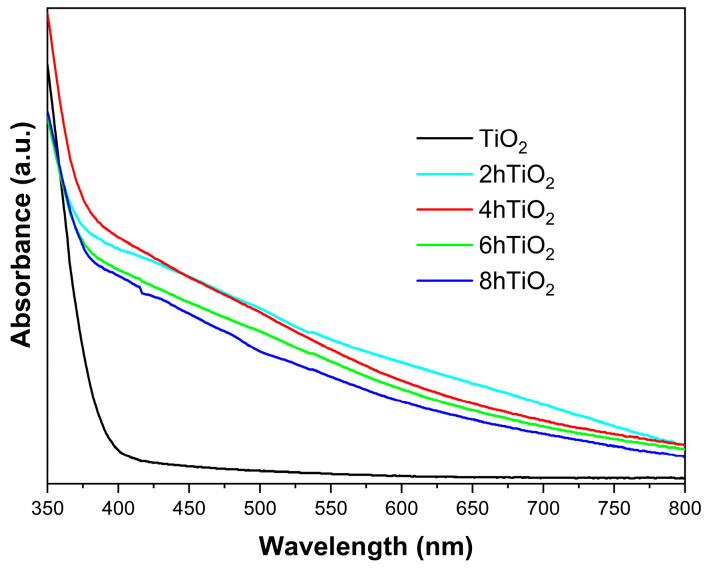
UV-vis absorption spectra for mesoporous TiO_2_ Nanospheres and the vacuum activated samples.

Figure 3 shows the solid-state UV-Vis absorption spectra of all five samples. The UV-Vis spectrum of the vacuum high-temperature treated samples (2hTiO_2_, 4hTiO_2_, 6hTiO_2_, and 8hTiO_2_) demonstrates an increase in absorption starting at 800 nm, indicating that these samples can absorb light in the visible light region. This shift toward longer wavelengths, known as a redshift, suggests that the treatment extends the light absorption range of TiO_2_ into the visible spectrum.

In contrast, the untreated pure TiO_2_ Nanospheres exhibit significant absorption only in the ultraviolet (UV) region with wavelengths shorter than 400 nm. The UV-Vis spectra qualitatively show that the vacuum high-temperature activation treatment significantly enhances the light absorption capability of TiO_2_ in the visible region. This indicates a notable improvement in the photosensitivity of the treated samples, likely due to the formation of defects or electronic structure modifications induced by the high-temperature treatment, which facilitate the absorption of visible light.

As shown in Figure 3, the Ti^3+^ self-doped samples exhibit a distinct inflection point around 400 nm, corresponding to the band-to-band transition characteristics of the material. Therefore, we propose that the Ti^3+^ self-doping does not alter the material’s bandgap. Instead, the continuous absorption in the 400–800 nm range is attributed to the formation of numerous sub-bandgap states induced by Ti^3+^, leading to continuous visible light absorption [35].

These results confirm that the vacuum high-temperature activation treatment effectively improves the photocatalytic properties of TiO_2_ by expanding its absorption spectrum into the visible light range, thereby increasing its potential for applications in solar-driven photocatalysis. The redshift observed in the treated samples is consistent with the enhancement of the material’s light-harvesting capability, which is crucial for improving the efficiency of photocatalytic processes under solar irradiation.

### 2.4. Kinetics of Photodegradation Analysis

**Figure 4:**
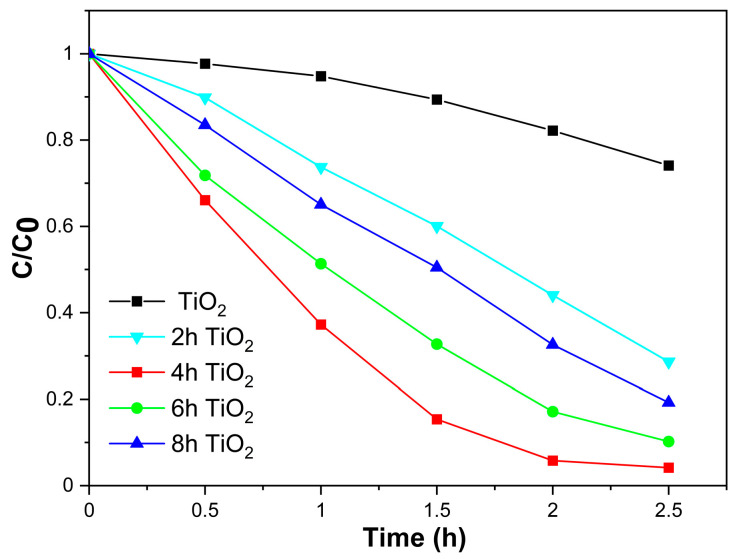
Kinetics of photodegradation of 20 mg L^−1^ Rhodamine B (RhB)by vacuum activated TiO_2_ under visible light irradiation for 2.5 h.

The photocatalytic activity of TiO_2_, 2hTiO_2_, 4hTiO_2_, 6hTiO_2_, and 8hTiO_2_ was evaluated by simulating sunlight using a xenon lamp, optical filters, and a condenser, with Rhodamine B (RHB) degradation as the test reaction. Figure 4 presents the degradation results for these samples over a 2.5 h period. The degradation rates of TiO_2_, 2hTiO_2_, 4hTiO_2_, 6hTiO_2_, and 8hTiO_2_ were 25.9%, 71.4%, 95.8%, 89.8%, and 80.7%, respectively. Notably, the 4hTiO_2_ sample exhibited the highest photocatalytic activity, with a degradation rate of approximately 95%, indicating its superior performance compared to the other samples.

Comparing the results, it is evident that all four treated samples (2hTiO_2_, 4hTiO_2_, 6hTiO_2_, and 8hTiO_2_) achieved degradation rates greater than 60% under visible light irradiation over 2.5 h, demonstrating that the vacuum high-temperature activation treatment significantly enhanced the photocatalytic efficiency of TiO_2_ for RHB degradation.

TiO_2_, as a wide band-gap semiconductor with a band gap of approximately 3.2 eV, primarily responds to UV light, leading to electron-hole separation. However, the observed photocatalytic activity of the treated samples under simulated visible light irradiation can be attributed to the sensitization effect of RHB. The enhanced photocatalytic activity under visible light is likely due to the extension of the TiO_2_ absorption spectrum into the visible region, as shown in the UV-Vis results, along with the possible formation of surface defects or dopant-induced changes that facilitate electron transfer and improve charge separation.

These findings highlight the effectiveness of the vacuum high-temperature treatment in enhancing the visible light photocatalytic activity of TiO_2_, making it a promising material for solar-driven photocatalysis applications.

### 2.5. Photocurrent Analysis

**Figure 5:**
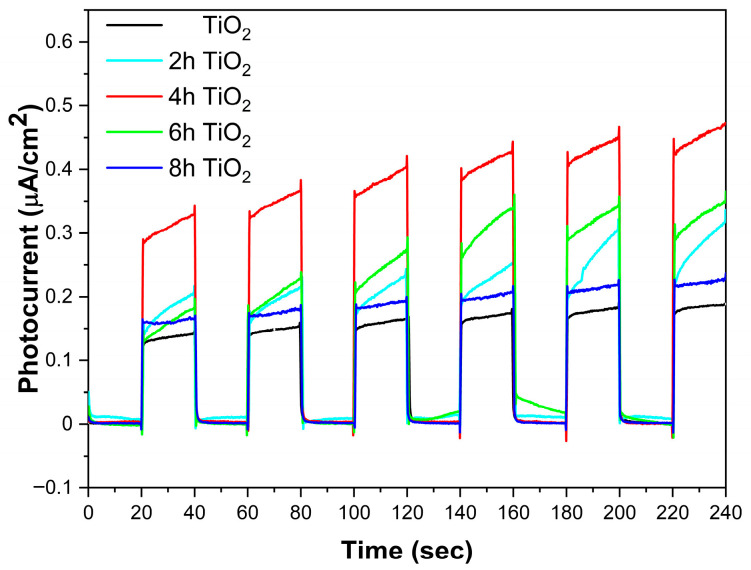
Photocurrent of TiO_2_, 2h TiO_2_,4h TiO_2_,6h TiO_2_ and 8h TiO_2_ under visible light (>400 nm) on/off cycles.

Figure 5 presents the photocurrent cycling comparison for TiO_2_, 2hTiO_2_, 4hTiO_2_, 6hTiO_2_, and 8hTiO_2_ under light and dark conditions. From the figure, it is evident that the photocurrent density of all five samples is approximately 0.13 μA·cm^−2^ under light/dark cycles. However, the photocurrent density of the TiO_2_ Nanospheres treated under vacuum high-temperature conditions is significantly enhanced. Among the treated samples, 4hTiO_2_ exhibited the highest photocurrent density, indicating that the vacuum high-temperature treatment has a notable effect on TiO_2_.

The increased photocurrent density for the treated TiO_2_ Nanospheres suggests that the high-temperature treatment promotes the separation of photogenerated electrons and holes within the material, which is essential for efficient photocatalytic processes. This improvement in charge carrier separation is likely due to the formation of surface defects or changes in the electronic structure induced by the vacuum treatment. The enhancement in photocurrent density further supports the hypothesis that the high-temperature treatment significantly improves the material’s ability to efficiently separate and transport charge carriers, which is critical for enhancing its photocatalytic performance under light irradiation.

The photocatalytic results indicate that the 4 h sample exhibits the best photocatalytic performance. As shown in Figure 5, the 4 h sample demonstrates the highest photocurrent density, suggesting that it generates more photogenerated charge carriers on its surface. We propose that the relatively short vacuum treatment time facilitates the release of an appropriate amount of oxygen and the formation of Ti^3+^ ions. These limited Ti^3+^ ions self-doped into the material, generating sub-bandgap states that enable charge carrier separation. According to Figure 3, these treated samples exhibit a band-to-band transition inflection point near 400 nm, without altering the fundamental band structure of TiO_2_. For the 6 h and 8 h treated samples, as the treatment time increases, a decrease in crystallinity is observed via XRD analysis. This may be due to the formation of an amorphous Ti_2_O_3_-like phase in localized regions caused by excessive Ti^3+^, which impedes effective charge transport. Additionally, photogenerated charge carriers can recombine at these defect sites, thereby reducing photocatalytic activity.

These results highlight the positive impact of the vacuum high-temperature treatment on the photoelectric properties of TiO_2_ Nanospheres, demonstrating that this process can effectively enhance charge separation and improve the photocurrent response, which is beneficial for applications in solar-driven photocatalysis.

### 2.6. SPV Analysis

**Figure 6:**
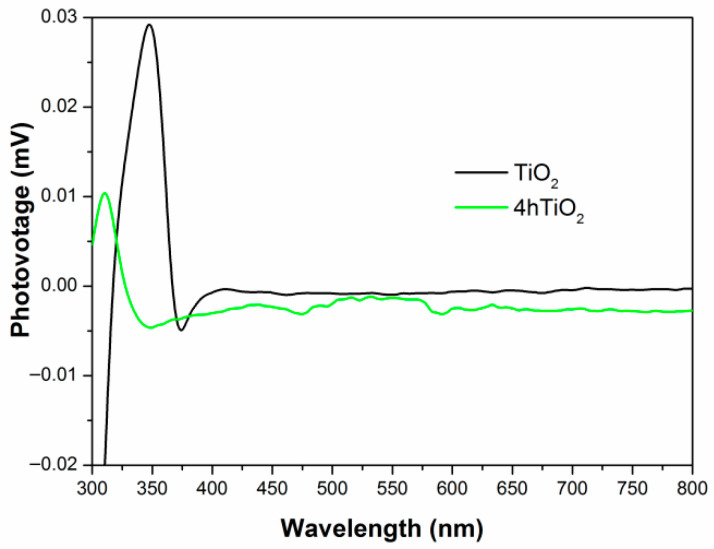
Surface photovoltage spectra.

Figure 6 shows the surface photovoltage (SPV) spectra for TiO_2_ and 4hTiO_2_. The SPV response of TiO_2_ Nanospheres is observed in the 300–380 nm range, while 4hTiO_2_ exhibits a response across the entire spectrum. This broadening of the SPV response range in 4hTiO_2_ can be attributed to the vacuum high-temperature treatment, which likely enhances the surface properties and promotes more effective charge separation.

The transmission electron microscopy (TEM) images of both TiO_2_ and 4hTiO_2_ indicate that their surfaces are relatively rough, which can lead to the formation of defect states at the grain boundaries. Additionally, the gaps between microcrystals could adsorb gas molecules, contributing to surface states. For 4hTiO_2_, the extended vacuum high-temperature treatment time reduces the crystallinity of the material, leading to an increase in lattice defects and impurities. These structural modifications enhance the formation of surface states, which are responsible for the extended SPV response observed across the entire spectral range.

The presence of defect states and surface states creates temporary intermediate energy levels within the band gap. Under light irradiation, charge transitions between the valence band, these intermediate states, and the conduction band generate the observed photocurrent response. The broad SPV response of 4hTiO_2_ indicates that the increased surface defects and impurities facilitate the generation of intermediate energy levels, promoting charge carrier excitation and improving the photocatalytic activity over a wider range of the electromagnetic spectrum.

These findings demonstrate that the vacuum high-temperature treatment significantly influences the surface properties and electronic structure of TiO_2_, leading to an enhanced photocatalytic performance due to the increased surface states and the associated charge carrier dynamics.

### 2.7. XPS Analysis

**Figure 7:**
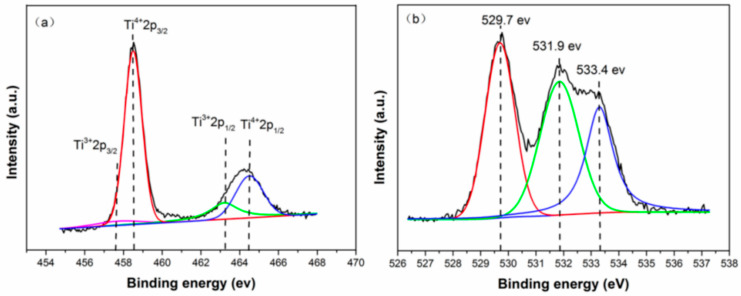
High-resolution XPS spectra of Ti 2p (**a**) and O 1s (**b**) for 4hTiO_2_.

Figure 7 shows the Ti 2p (a) and O 1s (b) X-ray photoelectron spectroscopy (XPS) spectra of the 4hTiO_2_ sample. The Ti 2p binding energies for pure TiO_2_ are typically observed around 464.5 eV and 459.0 eV [36]. However, in the 4hTiO_2_ sample, the presence of Ti^3+^ results in a shift in the binding energies to lower values [37]. The Ti 2p peak is complex, with four sub-peaks corresponding to Ti^3+^ 2p_3_/_2_ at approximately 456.8 eV, Ti^4+^ 2p_3_/_2_ at around 458.5 eV, Ti^3+^ 2p_1_/_2_ at approximately 463.2 eV, and Ti^4+^ 2p_1_/_2_ at around 464.5 eV [38,39]. As shown in Figure 7a, the intensity of the Ti^3+^ peak is relatively weak, indicating that the concentration of Ti^3+^ in the 4hTiO_2_ sample is low.

The O 1s binding energy of Ti-O bonds is typically in the range of 529.5 eV to 530.5 eV [40]. Figure 7b presents the O 1s XPS spectrum of the 4hTiO_2_ sample, where three distinct peaks are observed within this range. These peaks suggest that the oxygen is predominantly in the form of Ti-O bonds. The peak at 529.7 eV corresponds to the Ti-O bond, while the peak at 531.9 eV is characteristic of surface oxygen vacancies (O_v_) [41]. The weak peak at 533.4 eV corresponds to the hydroxyl (-OH) group [42], indicating the presence of -OH species on the sample surface.

The introduction of Ti^3+^ and oxygen vacancies (O_v_) in TiO_2_ creates localized states approximately 0.75–1.18 eV below the conduction band. These localized states enable TiO_2_ to absorb visible and infrared light. As the concentrations of Ti^3+^ and Ov increase, these localized states merge to form an intermediate energy band, which effectively narrows the band gap of TiO_2_ [43]. This modification in the electronic structure enhances the photocatalytic activity by extending the material’s absorption spectrum into the visible and infrared regions, thus improving its overall photocatalytic efficiency under solar irradiation.

These XPS results confirm the presence of Ti^3+^ and oxygen vacancies, which play a crucial role in improving the light absorption properties and photocatalytic performance of TiO_2_ under visible light. The formation of an intermediate energy band through the introduction of Ti^3+^ and Ov significantly contributes to reducing the band gap of TiO_2_, facilitating its use in solar-driven photocatalysis.

### 2.8. Mott-Schottky Analysis

**Figure 8:**
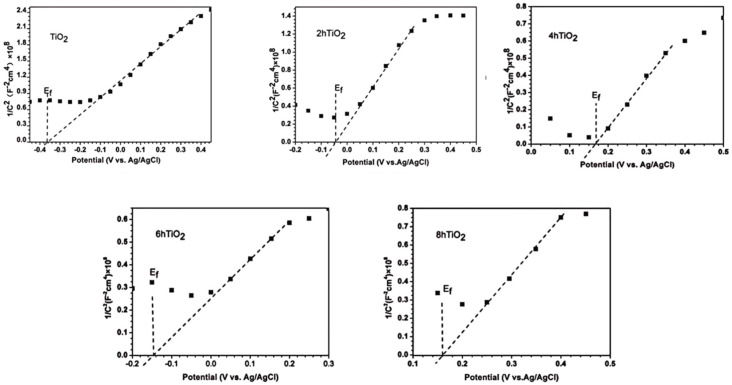
Mott–Schottky plots of TiO_2_, 2h TiO_2_,4h TiO_2_,6h TiO_2_ and 8h TiO_2_ in 0.2M Na_2_SO_4_ solution.

The flat band potential of a semiconductor can be determined from the Mott-Schottky plot, which reveals the type of semiconductor behavior. A positive slope in the Mott-Schottky curve indicates that the material is an n-type semiconductor, while a negative slope suggests it is a p-type semiconductor. Figure 8 presents the Mott-Schottky plots for TiO_2_, 2hTiO_2_, 4hTiO_2_, 6hTiO_2_, and 8hTiO_2_. According to the Mott-Schottky equation:

$$\frac{1}{C^{2}}=\frac{2}{e\varepsilon \varepsilon_{0}N_{A}}(E-E_{F}-\frac{kT}{e})$$

where C is the capacitance, e is the charge, ε is the dielectric constant, ε_0_ is the vacuum dielectric constant, N_A_ is the Avogadro constant, E is the applied voltage, and E_F_ is the flat band potential.

From the Mott-Schottky plots, the inverse capacitance squared (1/C^2^) is plotted against the applied voltage (E), which yields a linear relationship. The intersection of the curve with the x-axis corresponds to the flat band potential (approximately equal to the conduction band). As shown in Figure 8, the slope of the Mott-Schottky curves is positive, indicating that all the samples exhibit n-type semiconductor behavior. This result suggests that the vacuum high-temperature treatment does not alter the semiconductor type of TiO_2_ (Table 1).

### 2.9. Visible-Light Activation Mechanism of Brown TiO_2_

**Figure 9:**
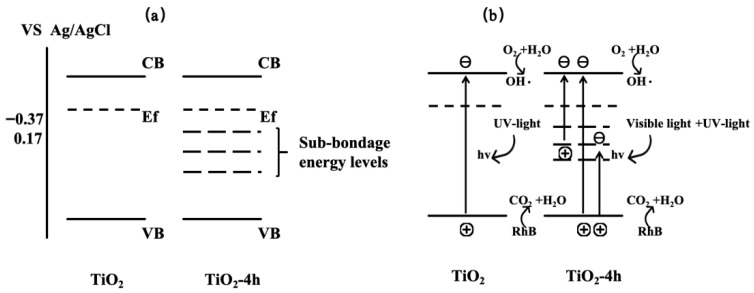
Visible-light activation mechanism of brown TiO_2_. (**a**) Band structures of pristine TiO_2_ and vacuum-treated TiO_2_. (**b**) Photocatalytic charge-transfer pathways of pristine and vacuum-treated TiO_2_, where sub-bandgap energy levels enable visible-light-induced charge excitation.

Furthermore, based on the Mott-Schottky theory, the flat band potentials (approximately equal to the Fermi level) for TiO_2_, 2hTiO_2_, 4hTiO_2_, 6hTiO_2_, and 8hTiO_2_ were approximately −0.37 V, −0.04 V, 0.17 V, −0.15 V, and 0.16 V vs. Ag/AgCl, respectively. These values demonstrate that the flat band potential shifts with the treatment time, which may be linked to changes in the electronic structure or the formation of surface defects during the vacuum treatment. The variation in the flat band potential suggests modifications in the electron density and electronic properties of the material, which can influence its photocatalytic performance. Based on the above results, the band structure of brown TiO_2_ can be qualitatively analyzed, as illustrated in Figure 9. After high-temperature vacuum treatment, the samples exhibit a lower Fermi level, indicating the formation of a greater number of sub-bandgap energy levels located deeper within the forbidden band between the valence band and the conduction band. Under visible-light irradiation, electrons can be excited from the valence band to these empty sub-bandgap states, or further promoted from these states to the conduction band, thereby generating photogenerated holes and reactive radicals that participate in the oxidation of RhB. This mechanism is consistent with the broad absorption band observed between 400 and 800 nm in the UV–vis absorption spectra. The detailed nature and internal structure of these sub-bandgap energy levels may be further elucidated in future work by first-principles calculations.

The photocatalytic activity of TiO_2_ can be significantly enhanced through Ti^3+^ self-doping and the introduction of oxygen vacancies (O_v_), which modify the material’s electronic structure. These defects introduce localized states within the bandgap, narrowing the TiO_2_ bandgap and enabling more efficient light absorption, especially in the visible region. Ti^3+^ ions reduce the recombination of photogenerated electron-hole pairs, as shown by Peiris et al. [44], facilitating charge carrier separation and increasing active sites for photocatalytic reactions.

Guo et al. [45] further emphasize that Ti^3+^ defects act as trapping sites for electrons, improving charge separation and enhancing photocatalytic efficiency in reactions such as pollutant degradation. Our findings show that the 4 h treated TiO_2_ sample exhibits the highest photocatalytic performance, with optimal Ti^3+^ and Ov concentrations. However, longer treatment times (6 h and 8 h) lead to decreased crystallinity (as seen in XRD), and excessive defect formation results in reduced photocatalytic efficiency due to the creation of amorphous Ti_2_O_3_-like phases that hinder charge transport.

In comparison to other TiO_2_ modifications like black TiO_2_, which also benefits from Ti^3+^ and Ov defects, our approach offers a simpler, more scalable method that avoids high-temperature sintering. The vacuum treatment method effectively controls defect formation, maintaining high crystallinity and enhancing photocatalytic performance, making it more suitable for practical applications.

In conclusion, the enhanced photocatalytic performance of our TiO_2_ samples is attributed to the synergistic effects of Ti^3+^ self-doping and oxygen vacancy formation, improving light absorption and charge separation for efficient photocatalysis.

## 3. Experiment

### 3.1. Materials and Methods

The chemical reagents used in this study are of analytical grade, and no additional purification was performed. The specific reagents and their sources are as follows: Titanium tetrabutoxide, Ethylene glycol, Propanol, Rhodamine B, Anhydrous ethanol, provided by Sigma-Aldrich (St. Louis, MO, USA).

Main instruments: X-ray diffractometer (XRD), Rigaku D/MAX-2500 (Rigaku Corporation, Tokyo, Japan), used for characterizing the crystal structure of ZnO. Transmission electron microscope (TEM), JEOL JEM-2100 (JEOL Ltd., Tokyo, Japan), was used to observe the sample’s morphology and particle size. X-ray photoelectron spectroscopy (XPS), PHI 5000 (Physical Electronics, Chanhassen, MN, USA), was used to analyze the sample’s surface chemical state.

### 3.2. Sample Preparation

#### 3.2.1. Sample Preparation for TiO_2_ Nanospheres

At room temperature, 25 mL of ethylene glycol was added to a round-bottom flask, followed by 1 mL of titanium tetrabutoxide (TBT). The mixture was vigorously stirred, and nitrogen gas was introduced for 10 min to expel air from the system. The reaction was sealed and stirred for 24 h. After the reaction, the mixture was quickly poured into a solution of 100 mL acetone and 1 mL deionized water, and stirred for approximately 10 min. The solution was then allowed to stand for about 16 h.

After aging, the white solid was collected via centrifugation. To remove residual organic impurities, the solid was washed five times with acetone and ethanol. The washed white solid was then dried in a convection oven.

For the hydrothermal treatment, 1 g of the dried sample was transferred to a 100 mL high-pressure reaction vessel, and 80 mL of ultra-pure water was added. The vessel was sealed and heated at 180 °C for approximately 4 h. After the reaction, the vessel was removed from the oven and allowed to cool to room temperature. The white precipitate was separated by centrifugation and washed multiple times with ultra-pure water and ethanol. The resulting white precipitate was dried, yielding the TiO_2_ Nanospheres, which were subsequently stored for further use.

#### 3.2.2. Vacuum High-Temperature Treatment of TiO_2_ Nanospheres

The TiO_2_ Nanospheres obtained from the synthesis process were placed in a vacuum drying oven. The temperature was gradually increased to 190 °C, and the vacuum pressure was set to 2 × 10^4^ Pa. The samples were subjected to high-temperature treatment for varying durations of 2 h, 4 h, 6 h, and 8 h, resulting in the formation of 2h TiO_2_, 4h TiO_2_, 6h TiO_2_ and 8h TiO_2_ samples, respectively.

### 3.3. Test of Photocatalytic

#### 3.3.1. Photocatalytic Degradation Under Visible Light

To evaluate the photocatalytic degradation performance, 25 mg of each sample was accurately weighed and placed in a photoreactor. Then, 20 mL of a 20 mg·L^−1^ Rhodamine B (RHB) aqueous solution was added. The photoreactor was wrapped with aluminum foil and stirred for 30 min to ensure proper mixing. After the stirring period, 1 mL of the supernatant was collected using a micropipette. The aluminum foil was removed, and a xenon lamp was switched on. A filter (wavelength < 400 nm) and a condenser were used to ensure that the incident light remained within the visible light range. The system was irradiated continuously, and 1 mL of the supernatant was sampled every 30 min. Four samples were sequentially withdrawn, labeled, and their concentrations were measured using a visible light spectrophotometer. The concentration data were used to generate C/C_0_ vs. time plots for analysis of the photocatalytic activity of the samples.

#### 3.3.2. Photocurrent Measurement Under Visible Light

For photocurrent measurements, 50 mg of each sample was placed in a sample bottle, and 1 mL of deionized water was added. The mixture was stirred with a magnetic stirrer until a paste-like consistency was achieved. A small amount of this paste was transferred using a pipette and applied onto an FTO (fluorine-doped tin oxide) glass substrate with dimensions approximately 5 mm × 5 mm. The coated FTO glass was allowed to dry at room temperature, forming the working electrode.

**Figure 10:**
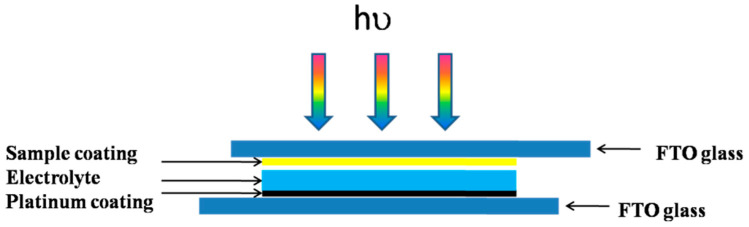
Schematic Diagram of the Photocurrent Measurement System.

The working electrode and a platinum counter electrode were securely clamped. A 1 mol/L I_3_^−^/I^−^ electrolyte solution was injected between the two electrodes using a 50 μL micropipette. The working electrode, which contained the sample, was positioned between the FTO conductive glass, while the reference electrode and counter electrode were attached to the platinum electrode. The system was exposed to light from a xenon lamp, with the light intensity adjusted to 100 mW·cm^−2^. A filter (wavelength < 400 nm) was used to ensure that the incident light was within the visible light range. The photocurrent was measured using the setup depicted in Figure 10.

#### 3.3.3. Measurement of Flat Band Potential

For the measurement of the flat band potential, 50 mg of each sample was weighed and placed into a sample bottle. Then, 1 mL of deionized water was added, and the mixture was stirred with a magnetic stirrer until a paste-like consistency was achieved. A small amount of the paste was transferred using a pipette and applied onto an FTO (fluorine-doped tin oxide) glass substrate with dimensions approximately 5 mm × 5 mm. The coated FTO glass was left to dry at room temperature to form the working electrode.

A platinum electrode was used as the counter electrode, and an Ag/AgCl electrode served as the reference electrode. The flat band potential was measured by placing the sample-coated FTO glass as the working electrode in the system, and the measurements were conducted under standard electrochemical conditions.

### 3.4. Material Characterization

X-ray powder diffraction (XRD) was used to analyze the diffraction patterns of the materials, providing information on their chemical composition and internal atomic or molecular structure (PANalytical, Almelo, The Netherlands). Material morphology was examined by field-emission scanning electron microscopy (FE-SEM) (Hitachi, Tokyo, Japan) and transmission electron microscopy (TEM) (JEOL Corporation, Tokyo, Japan).

## 4. Conclusions

In this study, TiO_2_ Nanospheres with a diameter of approximately 250 nm were synthesized via a hydrothermal method using tetrabutyl titanate as the precursor. The TiO_2_ nanosphere powder was then subjected to vacuum high-temperature treatment for 2, 4, 6, and 8 h. Transmission electron microscopy (TEM) characterization revealed that the vacuum high-temperature treatment did not alter the morphology or structure of the nanospheres, which remained loose and porous, thereby enhancing their ability to utilize solar light.

Photocatalytic degradation of Rhodamine B (RHB) under simulated visible light conditions showed that the 4hTiO_2_ sample exhibited the highest degradation rate among all the samples, indicating its superior photocatalytic performance. The flat band potential of 4hTiO_2_ was found to be 0.17 V vs. Ag/AgCl, corresponding to the highest conduction band position. X-ray photoelectron spectroscopy (XPS) analysis revealed that the 4hTiO_2_ sample, after high-temperature vacuum activation, contained Ti^3+^ and oxygen vacancies, which contributed to enhanced light absorption and the generation of a large number of photogenerated electrons and holes. These results suggest that the 4hTiO_2_ sample possesses improved photocatalytic water splitting capabilities, driven by its enhanced photosensitivity and the presence of surface defects. Therefore, vacuum high-temperature treatment effectively enhances the photocatalytic performance of TiO_2_ Nanospheres, making them promising candidates for solar-driven photocatalytic applications.

## Figures and Tables

**Table 1:** Flat band potential (E_fb_) values of TiO_2_ and its treated variants (2hTiO_2_, 4hTiO_2_, 6hTiO_2_, and 8hTiO_2_).

	TiO_2_	2h TiO_2_	4h TiO_2_	6h TiO_2_	8h TiO_2_
E_fb_(ev)	−0.37 ± 0.02	−0.04 ± 0.015	0.17 ± 0.02	−0.15 ± 0.02	0.16 ± 0.015

## Data Availability

The data that support the findings of this study are available from the corresponding authors upon reasonable request.
